# Multiscale dynamic mean field (MDMF) model relates resting-state brain dynamics with local cortical excitatory–inhibitory neurotransmitter homeostasis

**DOI:** 10.1162/netn_a_00197

**Published:** 2021-08-30

**Authors:** Amit Naskar, Anirudh Vattikonda, Gustavo Deco, Dipanjan Roy, Arpan Banerjee

**Affiliations:** Cognitive Brain Dynamics Lab, National Brain Research Centre, Manesar, Gurgaon, India; Cognitive Brain Dynamics Lab, National Brain Research Centre, Manesar, Gurgaon, India; Computational Neuroscience Research Group, Universitat Pompeu Fabra, Barcelona, Spain; Cognitive Brain Dynamics Lab, National Brain Research Centre, Manesar, Gurgaon, India; Cognitive Brain Dynamics Lab, National Brain Research Centre, Manesar, Gurgaon, India

**Keywords:** MDMF, Neurotransmitters, Metastability, Resting-state functional and structural connectivity, Network measures, Neurological disorders

## Abstract

Previous computational models have related spontaneous resting-state brain activity with local excitatory–inhibitory balance in neuronal populations. However, how underlying neurotransmitter kinetics associated with E–I balance govern resting-state spontaneous brain dynamics remains unknown. Understanding the mechanisms by virtue of which fluctuations in neurotransmitter concentrations, a hallmark of a variety of clinical conditions, relate to functional brain activity is of critical importance. We propose a multiscale dynamic mean field (MDMF) model—a system of coupled differential equations for capturing the synaptic gating dynamics in excitatory and inhibitory neural populations as a function of neurotransmitter kinetics. Individual brain regions are modeled as population of MDMF and are connected by realistic connection topologies estimated from diffusion tensor imaging data. First, MDMF successfully predicts resting-state functional connectivity. Second, our results show that optimal range of glutamate and GABA neurotransmitter concentrations subserve as the dynamic working point of the brain, that is, the state of heightened metastability observed in empirical blood-oxygen-level-dependent signals. Third, for predictive validity the network measures of segregation (modularity and clustering coefficient) and integration (global efficiency and characteristic path length) from existing healthy and pathological brain network studies could be captured by simulated functional connectivity from an MDMF model.

## Author Summary

How changes in neurotransmitter kinetics impact the organization of large-scale neurocognitive networks is an open question in neuroscience. Here, we propose a multiscale dynamic mean field (MDMF) model that incorporates biophysically realistic kinetic parameters of receptor binding in a dynamic mean field model and captures brain dynamics from the “whole brain.” MDMF could reliably reproduce the resting-state brain functional connectivity patterns. Further employing graph theoretic methods, MDMF could qualitatively explain the idiosyncrasies of network integration and segregation measures reported by previous clinical studies.

## INTRODUCTION

Resting-state spontaneous activity (Mitra, Snyder, Hacker, & Raichle, 2014; Raichle, & Mintun, 2006; Rogers, Morgan, Newton, & Gore, 2007; Vincent et al., 2007) is presently well established as a substrate to understand normative brain patterns and intrinsic functional organization of macroscopic brain network dynamics. Furthermore, resting-state brain dynamics and its departure from normative patterns are important markers for characterizing various neurological/neuropsychiatric symptoms (Pearlson, 2017; Zhou, Liu, Kwun, & Wang, 2017). Numerous studies have attempted to understand the underlying mechanisms that govern the spatiotemporal dynamics of large-scale resting-state networks (Deco & Jirsa, 2012; Deco, Ponce-Alvarez, et al., 2014; Demirtas et al., 2017; see also Cabral, Kringelbach, & Deco, 2017, for a review). All these studies predominantly utilized a class of dynamic mean field (DMF) models known as whole-brain computational models that have emerged as an important tool to link the healthy and pathological brain network dynamics with underlying change in excitation–inhibition (E–I) balance (Abeysuriya et al., 2018; Deco, Ponce-Alvarez, et al., 2014; Vattikonda, Surampudi, Banerjee, Deco, & Roy, 2016).

Previous studies using whole-brain DMF suggest that temporal correlations among brain areas observed in resting state emerge, while (a) the brain dynamics operates close to criticality that is further parametrized using a global scaling parameter depicting global coupling strength between interconnected brain areas and (b) the excitatory input currents are balanced using feedback inhibition control mechanisms maintaining the local homeostasis (Abeysuriya et al., 2018; Deco & Jirsa, 2012; Deco, Ponce-Alvarez, et al., 2014; Vattikonda et al., 2016).

Valuable insights can be obtained from such studies; for example, at the resting state, the human brain operates at maximum metastability (Deco, Kringelbach, Jirsa, & Ritter, 2017), which allows for exploration of the dynamic repertoire of the brain’s intrinsic functional configurations and gives rise to rich brain network dynamics (Abeysuriya et al., 2018; Breakspear, 2017). However, one important challenge is to understand how the interaction among physiological parameter(s) like excitatory and inhibitory neurotransmitter concentrations at the microscale shapes the spontaneous and structured resting-state dynamics of the brain. The problem is further compounded by the fact that brain parameters, such as the proportion of different neurons and correspondingly neurotransmitter types, such as glutamate, gamma-aminobutyric acid (GABA), with their associated synaptic properties cannot be manipulated independently in living systems to delineate their role in brain function (Prinz, 2008). Thus, physiologically realistic whole-brain network models are favorable candidates to overcome and manipulate experimentally inaccessible parameter spaces of the system (Breakspear, 2017).

GABA and glutamate are the two key inhibitory and excitatory neurotransmitters, respectively (Novotny, Fulbright, Pearl, Gibson, & Rothman, 2003), that are present all over the cortex (Duncan, Wiebking, & Northoff, 2014) and govern the excitatory–inhibitory (E–I) balance (Lauritzen, Mathiesen, Schaefer, & Thomsen, 2012). Several studies have demonstrated that GABA has significant association with blood-oxygen-level-dependent (BOLD) signals in a healthy human brain (Northoff, Duncan, & Hayes, 2010; Raichle, 2009). While interaction among inhibitory neurons and excitatory neurons is thought to have a direct influence on BOLD signals (Logothetis, Pauls, Augath, Trinath, & Oeltermann, 2001), the role of GABA and glutamate is poorly understood. Computational studies have shown how regulation of local E–I balance sculpts spontaneous resting-state activity of brain (Deco, McIntosh, et al., 2014; Deco, Ponce-Alvarez, et al., 2014), followed by a study from our group that has shown the importance of E–I balance in the context of functional recovery following different types of virtual lesions (Vattikonda et al., 2016) using whole-brain models. Some studies have further hypothesized that homeostatic deviations of excitatory–inhibitory balance is the key neuronal mechanism involved in pathological scenarios (Chiapponi, Piras, Piras, Caltagirone, & Spalletta, 2016). Hence, an extension of the modeling approach to understand the multiscale parameter space of interactions between neurotransmitter concentrations and neural population dynamics leading to understanding normative and pathological resting-state brain network dynamics can be of tremendous interest.

In order to link the E–I balance of DMF models to GABA-glutamate concentrations and propose a whole-brain model that bridges multiple scales of cellular organization, we chose the synaptic gating parameter in the DMF as an entry point. Synaptic gating from neurotransmitter binding to receptors has been explained theoretically via kinetic rate equation at the single synapse level by previous studies (Destexhe, Mainen, & Sejnowski, 1994a, 1994b). Here, we derive an averaged synaptic gating equation representing the combined synaptic gating of a population of neurons in a given brain area and combine them with a DMF model of excitatory/inhibitory neural populations (Deco, McIntosh, et al., 2014) that estimates total excitatory and inhibitory currents in a given brain region. Thus, we conceptualize a link between neurotransmitter concentration space and macroscale brain network dynamics by proposing a multiscale dynamic mean field (MDMF) model. An optimal parameter range of critical excitatory and inhibitory neurotransmitter concentrations (Deco et al., 2017) could be identified where the model generated resting-state functional connectivity (rs-FC) is proximal to empirical rs-FC. Additionally, our model is able to generate testable predictions about departure from normative values in neurotransmitter concentrations based on quantification of rs-FC using graph theoretic network measures of integration and segregation from a large number of existing pathological studies (Jiang, Li, Chen, Ye, & Zheng, 2017; Sun et al., 2016; Wang et al., 2014; Yasuda et al., 2015).

## MATERIALS AND METHODS

### Multiscale Dynamic Mean Field Model

**Figure 1.**
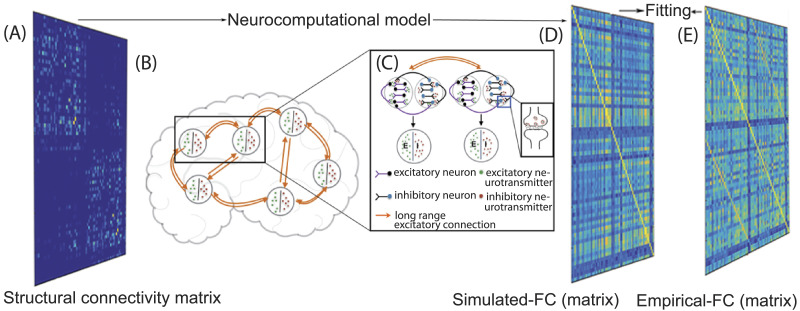
Multiscale dynamic mean field (MDMF) model setup. (A) Averaged anatomical connectivity or structural connectivity matrix of healthy human brains. (B) Whole-brain network composed of interconnected nodes. (C) Local networks, represented by nodes or cortical areas, with each separate node consisting of excitatory (purple) and inhibitory (black) neurons with recurrent inhibitory–inhibitory, excitatory–excitatory, inhibitory–excitatory, and excitatory–inhibitory connection; all nodes are discrete and interconnected with long-range excitatory neurons (orange line). Each brain area is represented as a pool of excitatory (E, green) and inhibitory (I, red) neurotransmitters. In inset image, presynaptic neuron releases neurotransmitter in the synaptic cleft, and it binds with postsynaptic receptors. (D) Model-generated FC matrix, and (E) its fit or similarity with empirical FC.

Previous studies have shown that the dynamic mean field approach (Deco, McIntosh, et al., 2014; Deco, Ponce-Alvarez, et al., 2014) is able to capture the rs-FC using feedback inhibition control to maintain E–I balance and constrain network dynamics with empirical structural connectivity (SC) extracted from the density of white matter fiber tracts connecting brain areas. One can go further to identify the role of local E–I homeostasis in shaping up rs-FC when structural connections are perturbed (Vattikonda et al., 2016). On the other hand, at neurotransmitter level, the kinetic model of receptor binding (Destexhe et al., 1994a, 1994b) relates the neurotransmitter concentration changes to the synaptic gating variable. Thus, a multiscale model should capture the synaptic gating dynamics, which are linked to neurotransmitter concentration kinetics and further play a key role in generation of excitatory–inhibitory currents in a local population of neurons (mean field). Subsequently, we can relate the electric mean field activity in an area to the BOLD activity by the hemodynamic model (Friston, Harrison, & Penny, 2003; Friston, Mechelli, Turner, & Price, 2000). Each cortical region can be modeled as a pool of excitatory and inhibitory neurons with recurrent excitatory–excitatory, inhibitory–inhibitory, excitatory–inhibitory, and inhibitory–excitatory connections, and coupled with neurotransmitters GABA and glutamate via NMDA (N-methyl-D-aspartate) and GABA synapses, respectively (Figure 1). Long-range connections are modeled as excitatory connections between excitatory pools of each region. Long-range inputs are also scaled according to connection strength between regions derived from fiber densities computed from diffusion tensor imaging data. Excitatory population in an area receives the following input currents: recurrent inhibitory currents, recurrent excitatory currents, long-range excitatory currents from excitatory populations in all other areas, as well as external currents. However, the spatial variability of the neurotransmitter concentration has not been considered, so the values of glutamate and GABA concentration are identical in all brain areas for a simulation run.

The study of Destexhe et al. (1994a) assumed that occurrence of neurotransmitter release at synaptic cleft can be conceptualized as a pulse following the arrival of an action potential at the presynaptic terminal. Accordingly, we can consider that *R* + *T* ⇄ *TR* represents a chemical kinetic reaction where *T*, *R*, and *TR* are the neurotransmitters released in the synaptic cleft, unbound receptors, and bound receptors of postsynaptic neuron, respectively. Subsequently, neurotransmitter release can be captured in the rate equation that describes the dynamics of probability of open channels of a specific neurotransmitter (synaptic gating variable). The rate of change of synaptic gating variable for a neuron (*s*) is equivalent to the proportion of bound receptors in the postsynaptic neuron and can be expressed as neural response function (*δ*-spikes) scaled by maximal neurotransmitter concentration and the probability of channels being closed (1 − *s*) plus the contribution of leaky synaptic gating and the backward rate constant (Destexhe et al., 1994b):

$$\frac{ds}{dt}=-\beta s+\alpha \left[T_{\max}\right]\left(1-s\right)\sum_{t_{k}}\delta \left(t-t^{f}\right),$$ (1)

where, $\frac{ds}{dt}$ represents the rate of change in the synaptic gating variable, *t*^*f*^ are the spike times, and [*T*_max_] is maximun neurotransmitter concentration. *α* and *β* are the forward and backward rate constants of binding of neurotransmitters released into synaptic cleft from a presynaptic neuron onto receptors of postsynaptic neuron. Thus, *α* contributes to the bounded receptor buildup in postsynaptic neuron, while the *β* captures the leaky neurotransmitter loss to the cleft over the membrane.

At the population level, the average synaptic gating variable (*S*) can be computed for excitatory and inhibitory populations by taking the mean of gating variables across all subpopulations, *S* = 〈*s*〉. Assuming *α* and *β* do not change over an area as well as with time and firing rate, *r* = 〈∑_*tk*_ *δ*(*t* − *t*_*k*_)〉, the equation for average synaptic gating variable becomes

$$\frac{dS}{dt}=-\beta S+\alpha \left[T_{\max}\right]\left(1-S\right)r.$$ (2)

Previously Deco, McIntosh, et al. (2014) demonstrated that conductance-based models over an entire population of neurons can be used to derive the current-based field models such as a DMF model, capturing the input excitatory and inhibitory currents to an E–I population. Subsequently, combining a DMF model (Deco, McIntosh, et al., 2014; Deco, Ponce-Alvarez, et al., 2014) and an average kinetic model of receptor binding (Destexhe et al., 1994a, 1994b) that incorporates neurotransmitter concentrations (parameters) in synaptic gating dynamics (Equation 2), we propose the multiscale dynamic mean field model. Here, dynamics of inhibitory GABA-synaptic gating and excitatory NMDA-synaptic gating are governed by GABA and glutamate concentrations *T*_gaba_ and *T*_glu_, respectively. Thus, our implicit assumption is that the GABA and glutamate concentrations over a population are representative of the saturation achieved at several synaptic clefts simultaneously as per as BOLD timescale, the eventual empirical observation we are interested in, in this study. Earlier studies have shown that saturation of glutamate concentrations in synaptic clefts occur relatively fast in the order of a few milliseconds (G. Liu, Choi, & Tsien, 1999). The current-based MDMF model is described by the following coupled nonlinear stochastic differential equations:

$$I_{i}^{\left(E\right)}=W_{E}I_{0}+w_{+}J_{\text{NMDA}}S_{i}^{\left(E\right)}+{GJ}_{\text{NMDA}}\sum_{j}C_{ij}S_{j}^{\left(E\right)}-J_{i}S_{i}^{\left(I\right)}.$$ (3)

$$I_{i}^{\left(I\right)}=W_{I}I_{0}+J_{\text{NMDA}}S_{i}^{\left(E\right)}-S_{i}^{\left(I\right)}.$$ (4)

$$r_{i}^{\left(E\right)}=\left(a_{E}I_{i}^{\left(E\right)}-b_{E}\right)/\left(1-\exp^{\left(-d_{E}\left(a_{E}I_{i}^{\left(E\right)}-b_{E}\right)\right)}\right).$$ (5)

$$r_{i}^{\left(I\right)}=\left(a_{I}I_{i}^{\left(I\right)}-b_{I}\right)/\left(1-\exp^{\left(-d_{I}\left(a_{I}I_{i}^{\left(I\right)}-b_{I}\right)\right)}\right).$$ (6)

$$\frac{{dS}_{i}^{E}\left(t\right)}{dt}=-\beta^{E}S_{i}^{E}+\alpha^{E}\left(1-S_{i}^{E}\right)T_{glu}r_{i}^{E}+\sigma v_{i}\left(t\right).$$ (7)

$$\frac{{dS}_{i}^{I}\left(t\right)}{dt}=-\beta^{I}S_{i}^{I}+\alpha^{I}\left(1-S_{i}^{I}\right)T_{\text{gaba}}r_{i}^{I}+\sigma v_{i}\left(t\right).$$ (8)

**Figure 2.**
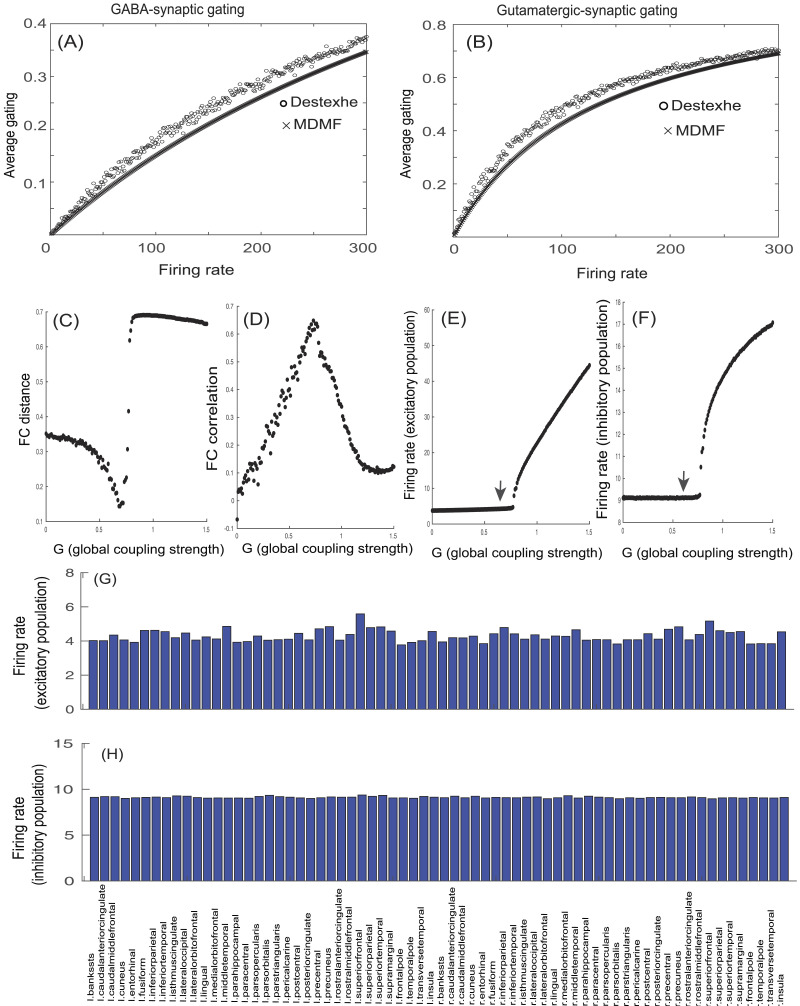
Comparison of MDMF model prediction of synaptic gating variables at steady state and the gating kinetics model proposed by Destexhe et al. (1994b). Model-generated average (A) GABA- or (B) NMDA-synaptic gating as a function of population mean firing rate (dotted lines) closely approximates the model proposed by Destexhe et al. (1994b) (lines with empty circles). (C) FC distance is examined as a function of *G*; each point represents FC distance for each *G* value. (D) FC correlation is examined as a function of *G*; each point represents FC correlation for each *G* value. For *G* = 0.69, FC distance is found to be minimum, whereas FC correlation is found to be maximum. (E) Average firing rate of excitatory population as a function of *G*; each point represents average firing rate of excitatory population for a specific *G* value. For *G* = 0.69, average firing rate of excitatory population is ∼4 Hz (represented by black arrow). (F) Mean firing rate of inhibitory population as a function of *G*; each point represents mean firing rate of inhibitory population for a specific *G* value. For *G* = 0.69, average firing rate of inhibitory population is ∼9 Hz (denoted by black arrow). (G) Firing rate of excitatory populations of all 68 brain areas (Desikan-Killiany atlas) is shown for *G* = 0.69. (H) Firing rate of inhibitory populations of all 68 cortical areas is shown for *G* = 0.69.

Equations 3–6 are identical to the DMF model proposed in Deco, Ponce-Alvarez, et al. (2014) and Deco, McIntosh, et al. (2014) where, $I_{i}^{\left(E\;\text{or}\;I\right)}$ is the input current to the area *i*, where superscripts I and E represent inhibitory and excitatory populations, respectively. Firing rates of excitatory and inhibitory populations of an area are given by $r_{i}^{\left(E\right)}$ and $r_{i}^{\left(I\right)}$, respectively. $S_{i}^{\left(E\right)}$ and $S_{i}^{\left(I\right)}$ are average excitatory and inhibitory synaptic gating variables of an area *i*, respectively. Effective external input current, *I*_0_, is scaled by *W*_I_ and *W*_E_ for inhibitory and excitatory populations. *C*_*ij*_ represents anatomical connectivity strength, derived from diffusion imaging, scaling long-range excitatory currents from region *j* to region *i*. Since recurrent excitatory currents are already taken into account while computing the input current to an excitatory population, all the diagonal elements *C*_*ii*_ are set to zero in the SC matrix. Excitatory synaptic coupling strength is given by *J*_NMDA_, while *J*_*i*_ denotes synaptic coupling strength from inhibitory to excitatory subpopulation. *v*_*i*_ in Equations 7 and 8 is uncorrelated standard Gaussian noise with standard deviation, *σ* = 0.001 nA. *G* represents global coupling strength scaling long-range excitatory connections in Equation 3 and is a free parameter in MDMF that is optimally fitted to describe empirical data following the earlier approach of Deco, Ponce-Alvarez, et al. (2014). The metrics for optimization that we use in this article are the rs-FC and the firing rate of neural populations in individual brain areas. In other words, for a fixed glutamate and GABA concentration that captures the healthy brain, we have selected the *G* value for which simulated rs-FC − empirical rs-FC correlation is maximum, while maintaining a firing rate ∼3–4 Hz (see Figure 2; Bittner et al., 2017). Nonetheless, theoretically multiple combinations of *G*, GABA, and glutamate may provide good correspondence between simulated and empirical rs-FC. Hence, we have examined multiple combinations *G*, GABA, and glutamate, that show good correspondence between simulated and empirical rs-FC, to show over a range of chosen *G* the overall pattern of results does not change (refer to the Supporting Information for more details). The balance between excitatory and inhibitory currents to a neuron can be established and maintained by inhibitory synapses using plasticity rules (Vogels, Sprekeler, Zenke, Clopath, & Gerstner, 2011). However, at the mean field level the biological complexity involved in the balance of dynamics between excitatory and inhibitory fields can be captured grossly using the mathematical implementation of the inhibitory plasticity rule (Hellyer, Jachs, Clopath, & Leech, 2016).

$$\frac{{dJ}_{i}\left(t\right)}{dt}=\gamma r_{i}^{I}\left(r_{i}^{E}-\rho \right).$$ (9)

Equation 9 describes the dynamics of *J*_*i*_, which is an inhibitory plasticity rule representing changes in *J*_*i*_ (synaptic weight) to ensure that inhibitory current clamps the excitatory current of a population, thereby maintaining a homeostasis. Homeostasis is achieved with *J*_*i*_ dynamics such that the firing rate of the excitatory population is maintained at the target firing rate *ρ* = 3 Hz, and *γ* is the learning rate in Equation 9. The chosen target firing rate is the firing rate observed when the inhibitory and excitatory currents are matched.

Synaptic activity of each area is used as input to the hemodynamic model (Friston et al., 2003; Friston et al., 2000) to generate the resting-state BOLD responses of each brain area. Differential equations are solved numerically using Euler–Maruyama method with time step of 0.1 ms, and all simulations are performed in MATLAB. All the parameters used for the simulation are given in Table 1. Simulations were carried out across glutamate (0.1 to 15 mmol) and GABA (0.1 to 15 mmol) concentration regimes that span the parameter space reported from healthy and diseased brains. However, to highlight an optimized range of neurotransmitter concentrations where normal resting-state brain function can be defined, we relate to empirical reports of neurotransmitter concentration (Govindaraju, Young, & Maudsley, 2000; Hu, Chen, Gu, & Yang, 2013).

**Table 1.** Parameters used to simulate MDMF mode

*I*_0_	0.382 *nA*	Deco, Ponce-Alvarez, et al. (2014)	*α* ^E^	0.072 *ms*^−1^ *mM*^−1^	Destexhe et al. (1994b)
*J*_NMDA_	0.15	Deco, Ponce-Alvarez, et al. (2014)	*β* ^E^	0.0066 *ms*^−1^	Destexhe et al. (1994b)
*W*_E_	1.0	Deco, Ponce-Alvarez, et al. (2014)	*α* ^I^	0.53 *ms*^−1^ *mM*^−1^	Destexhe et al. (1994b)
*W*_I_	0.7	Deco, Ponce-Alvarez, et al. (2014)	*β* ^I^	0.18 *ms*^−1^	Destexhe et al. (1994b)
*w*_+_	1.4	Deco, Ponce-Alvarez, et al. (2014)	*σ*	0.001 (*nA*)	Deco, McIntosh, et al. (2014)
*a*_I_	615 (n*C*^−1^)	Deco, Ponce-Alvarez, et al. (2014)	*G*	0.69	
*b*_I_	177 (Hz)	Deco, Ponce-Alvarez, et al. (2014)	*γ*	1	
*d*_I_	0.087 (s)	Deco, Ponce-Alvarez, et al. (2014)	*ρ*	3 Hz	Deco, Ponce-Alvarez, et al. (2014)
*a*_E_	310 (n*C*^−1^)	Deco, Ponce-Alvarez, et al. (2014)	*d*_E_	0.16 (s)	Deco, Ponce-Alvarez, et al. (2014)
*b*_E_	125 (Hz)	Deco, Ponce-Alvarez, et al. (2014)	*d*_i_	0.087 (s)	Deco, Ponce-Alvarez, et al. (2014)

### Validation on Empirical Data

For validation, MDMF units are placed in realistic cortical locations using structural connectivity matrix obtained from empirical data. Subsequently, rs-FCs from BOLD time series are computed using the MDMF approach from the same group of subjects and compared quantitatively with empirical rs-FC employing two different parcellation schemes for reliability. Further, global and local graph theoretic network measures from our model data are qualitatively compared with empirical observations previously reported in literature for understanding the pathological scenarios. The dataset used in this study is collected from the Cambridge Centre for Ageing and Neuroscience (Cam-CAN), University of Cambridge, UK, that uses a 3T Siemens TIM Trio scanner with a 32-channel head coil (voxel size 3 × 3 × 4.4 mm; Shafto et al., 2014).

### Generation of SC Matrix

Empirical diffusion-weighted imaging (DWI) data for our study are a randomized subsample of 40 healthy participants (19 males, age range 23–38; 21 females, age range 18–34) from a sample size of 175 volunteers in the age range of 18 to 40 years of the Cam-CAN dataset. We selected a focused age range to avoid age-related variability affecting interpretation of our observations. T1 anatomical images, diffusion-weighted images, gradient vectors, and gradient values are also obtained. The empirical SC matrix is generated following seed region of interest (ROI) selection, tracking, and aggregation of generated tracks by using an automated pipeline as proposed by Schirner, Rothmeier, Jirsa, Mcintosh, and Ritter (2015). High-resolution T1 anatomical images are used to create segmentation and parcellation of cortical gray matter using FreeSurfer’s (https://surfer.nmr.mgh.harvard.edu/) *recon-all* function.

For each subject, the following major steps are carried out: motion correction, skull stripping, removal of non-brain tissue, intensity normalization, brain mask generation, cortical reconstruction, cortical tessellation generating white matter (WM), gray matter (GM) and GM-pia interface surface-triangulations, WM segmentation, and probabilistic atlas-based cortical parcellation. WM–GM interface is employed as seeding and termination masks for diffusion-weighted magnetic resonance imaging (MRI) tractography. Along with the eddy current correction, geometric distortions correction, intervolume subject motion correction, bias-field correction, and denoising using MRtrix, the b0 image is linearly registered to the subject’s anatomical T1-weighted image thereby transforming the high-resolution mask volumes from the anatomical space to the subject’s diffusion space (via FSL’s flirt boundary-based registration).

Generated masks from WM segmentations are used to terminate tracks immediately when they leave WM. Moreover, MRtrix (https://www.mrtrix.org/) supported subvoxel tractography and tractography masks are generated from FreeSurfer’s high-resolution parcellation schemes. In this step, the diffusion tensor (so the diffusion ellipsoid) for each voxel is calculated and stored in image volumes; on this basis an eigenvector map and fractional anisotropy are computed and masked by the binary WM mask, which is created earlier. For the estimation of fiber-response function, the mask containing high-anisotropy voxels are calculated. Constrained spherical deconvolution algorithm that uses previously estimated fiber-response function computes fiber orientation distribution for each voxel in WM.

Cortical gray matter parcellation into 34 ROIs in each hemisphere is undertaken following Desikan-Killiany parcellation (Desikan et al., 2006) and to test robustness on the Destrieux brain atlas (Destrieux, Fischl, Dale, & Halgren, 2010) that parcellates each hemisphere into 75 ROIs. To estimate connection strength (value ranging from 0 to 1) between each pair of ROIs, probabilistic tractography algorithm is used to estimate how one ROI can influence the other in cortical GM parcellation. The pipeline aggregates generated tracks to estimate the structural connectome for each individual subject. The normalized weighted connection counts used here contain only distinct connections between each pair of regions.

### Generation of rs-FC Matrix

The major steps involved in preprocessing of image volumes using the CONN toolbox (https://web.conn-toolbox.org/) and SPM12 (https://www.fil.ion.ucl.ac.uk/spm/) are as follows: realignment, interframe motion correction and unwarping, slice timing correction, coregistration of the functional images to their respective anatomical MRI images, structural segmentation and spatial normalization, and functional spatial normalization (Whitfield-Gabrieli & Nieto-Castanon, 2012). For scans that are affected by movement-related artifacts, a motion and outlier scrubbing step is performed using the Artifact Detection Tools software in the CONN toolbox to correct those confounding effects, following which the functional images are smoothed using a Gaussian kernel of 8 mm full-width at half maximum. Here, T1-weighted images are segmented into WM, GM, and cerebrospinal fluid (CSF) areas. The temporal time series that characterizes the estimated subject motion (three rotation and three translation parameters, plus another six parameters representing their first-order temporal derivatives), in addition to signals emanating from the subject-specific WM mask and CSF mask, are used as temporal covariates. Those are removed using linear regression from the BOLD signal, and finally a band-pass filter (0.01 Hz < f < 0.10 Hz) is applied to get the residual BOLD time series.

Following the preprocessing of image volumes and subject-wise coregistration of functional images with T1-anatomical images, two different parcellation schemes were employed: 68 cortical areas (Desikan et al., 2006) and 150 cortical areas (Destrieux et al., 2010). FMRI data were collected from all participants with their eyes closed while remaining awake for 9 min, 20 s. For each participant, resting-state fMRI scans were acquired at 261 time points with TR = 1.97 s.

For each of the parcellation schemes, a representative BOLD signal is computed by calculating the mean of the BOLD signals from all the voxels in the corresponding ROI. Pairwise Pearson correlation coefficients were computed among ROIs from the z-transformed BOLD time series to generate the rs-FC matrix for each subject. Thus, the generated rs-FC matrix represents correlation of the BOLD activity between brain regions.

### Comparing Empirical and Simulated FC, Metastability

Prediction of empirical rs-FC was validated using the Frobenius norm of the difference between the simulated and empirical rs-FC matrices. Thus, FC distance (FCD) for one combination of glutamate and GABA is defined as the following:

$$FCD=\frac{1}{N}\sqrt{\sum_{i=1}^{N}\sum_{j=1}^{N}{\left(\text{empirical}\;FC\left(i,j\right)-\text{simulated}\;FC\left(i,j\right)\right)}^{2}}.$$ (10)

#### Using the measure of metastability to define optimal parameter space.

Metastability measures the tendency to deviate from stable manifolds in a dynamical system. For functional brain networks, a stable manifold may be the vector space spanned by the phase-locking indices, which captures synchronization among nodes. Earlier research has proposed that metastability is an important measure to describe information processing in a resting-state brain (Alderson, Bokde, Kelso, Maguire, & Coyle, 2018; Deco et al., 2017; Naik, Banerjee, Bapi, Deco, & Roy, 2017); more specifically, maximal metastability can be interpreted as a regime of maximal functional network switching (Deco et al., 2017). Hence, we seek to identify the parameter ranges of GABA/glutamate concentrations for which metastability computed from simulated and empirical BOLD time series were matched.

In the present study, metastability is measured using the standard deviation of the Kuramoto order parameter across time (Sahoo, Pathak, Deco, Banerjee, & Roy, 2020; Vasa et al., 2015). Kuramoto order parameter captures the degree of synchronization among a bunch of oscillators, and is defined by the following equation:

$$R\left(t\right)=\left|\sum_{k=1}^{n}e^{i\phi_{k}\left(t\right)}\right|/n,$$ (11)

where *ϕ*_*k*_(*t*) denotes the phase of each narrowband BOLD signal (band-passed at 0.03–0.06 Hz) at node *k*, and *n* is the total number of nodes. Hilbert transform was used to compute the instantaneous phase *ϕ*_*k*_(*t*) of each narrowband signal *k* using MATLAB function *hilbert.m* (Sahoo et al., 2020; Vasa et al., 2015). Earlier findings report that the narrow frequency bands have been mapped to the gray matter, and it has been found to be functionally relevant compared with other frequency bands (Achard, Salvador, Whitcher, Suckling, & Bullmore, 2006; Deco et al., 2017).

Order parameter *R*(*t*) measures the degree of synchronization of the *n* oscillating nodes at the global level. If *n* phases are uniformly distributed, then *R* takes on value ∼ 1/*n*, whereas if phases are perfectly synchronized over a time period, then <*R*(*t*)> = 1. Thus, the standard deviation of order parameter over time, $\sqrt{\langle {\left(R\left(t\right)-\langle R\left(t\right)\rangle \right)}^{2}\rangle }$, estimates the tendency of the system to deviate further away from the synchronization manifold over time and can qualify as a measure for metastability. Standard deviation of order parameter is calculated from both empirical resting-state fMRI signals and from simulated BOLD signals.

### Graph-Based Metrics to Assess Network Topologies

Adjacency matrices are constructed from rs-FC for applying graph theoretical metrics in the following steps. For network construction, only positive correlations of rs-FC are considered in the current study, and a proportional thresholding approach is used to compute the connectivity matrix (van den Heuvel et al., 2017). Although choosing sign of correlation for construction of graphs is a matter of debate (Fornito, Zalesky, & Breakspear, 2013), the earlier study by van den Heuvel et al. (2017) reports that no significant changes were found in overall FC while using the absolute value of correlation or taking only positive correlations, but low correlation thresholds can lead to spurious results. In a proportional thresholding approach, the number of edges remains the same for all the connectivity matrices (van den Heuvel et al., 2017). The number of edges to be preserved in the connectivity matrix solely depends on what value is assigned to the proportion of strongest weight (PSW). Once PSW is fixed, the same number of edges were considered for each connectivity matrix; in the current study PSW = 0.25 is used for proportional thresholding. Finally, proportional thresholded rs-FC matrices are used to generate binary adjacency matrices (setting all surviving connections to 1 and other connections to 0) for the various connection densities. Although there is no consensus in the literature on what specific proportional threshold should be used for the network construction, a range of 5% to 40% proportional thresholds have been reported (Fornito, Zalesky, & Bullmore, 2010). Brain Connectivity Toolbox is used to compute all the graph-based measures that quantify segregation and integration in information processing among brain areas, as described below. In addition, we have examined various PSW selections that are higher and lower than PSW = 0.25, which didn’t qualitatively change the pattern of results (see the Supporting Information).

### Integration Measures

#### Global efficiency.

Quantifies efficient exchange of information across the entire network (Wang, Zuo, & He, 2010). Global efficiency of graph Δ is denoted by the following formula:

$$E_{\text{glob}}\left(\Delta \right)=\frac{1}{n\left(n-1\right)}\sum_{i\neq j\in \Delta }\frac{1}{d_{ij}},$$ (12)

where *i* and *j* represent vertices in the graph, *n* is number of vertices, and *d*_*ij*_ is the shortest path length between node *i* and node *j* in Δ.

#### Characteristic path length.

Quantifies average shortest path length between all pairs of nodes in the network and measures efficiency of information transfer in a network.

### Segregation Measures

#### Clustering coefficient (*C*_p_).

*C*_p_ of a network is defined as the average of the clustering coefficients over all nodes in the network, where the clustering coefficient *C*_*i*_ of a node *i* is calculated using the following equation:

$$C\left(i\right)=\frac{2E}{k_{i}}\left(k_{i}-1\right),$$ (13)

where *E* represents the number of connections among the node *i*’s neighbors and *k*_*i*_ is the degree of node *i*.

#### Local efficiency.

Quantifies how well a network can exchange information when a node is removed.

$$E_{\text{local}}\left(\Delta \right)=\frac{1}{n}\sum_{i\in \Delta }E_{\text{glob}}\left(\Delta_{i}\right).$$ (14)

*E*_glob_(Δ_*i*_) is the global efficiency of subgraph Δ_*i*_, which is composed of the immediately adjacent neighbors of node *i*.

#### Modularity.

Quantifies the degree to which a network may be subdivided into clearly delineated groups; it is defined as the following:

$$Q=1/2l\sum_{ij}\left(A_{i,j}-\frac{k_{i}k_{j}}{2l}\right)\delta \left(s_{i},s_{j}\right),$$ (15)

where *l* is the total number of edges; *s*_*i*_ denotes the community to which *i* is assigned; *δ*(*s*_*i*_, *s*_*j*_) is 1, if *s*_*i*_ = *s*_*j*_, and 0 otherwise; *k*_*i*_ and *k*_*j*_ are the degree of nodes; and *A*_*i*,*j*_ is the number of edges between vertices *i* and *j*.

## RESULTS

### Whole-Brain MDMF Model Predicts rs-FC

The architecture of the MDMF model consisting of cortical areas or nodes that are interconnected with structural connections is represented in Figure 1A. The whole-brain network (Figure 1B) with long-range excitatory projections among distributed brain areas contribute to resting-state brain activity. By construction, averaged synaptic gating is scaled with releases of neurotransmitter, and neurotransmitter released in the synaptic cleft is shown in the inset image of Figure 1C. Each cortical area is described as a pool of excitatory and inhibitory populations with recurrent excitatory–excitatory, inhibitory–inhibitory, excitatory–inhibitory, and inhibitory–excitatory connections, whereas long-range connections are modeled as excitatory connections between excitatory populations of each region (Figure 1C). Synaptic gating depends on neurotransmitter released in the synaptic cleft, hence average synaptic gating is governed with mean release of neurotransmitter in a node, since there is no spatial distinction of cleft from the node in the MDMF model. So, the ensemble activity of each cortical area is the outcome of E–I neurotransmitter homeostasis (Figure 1C). MDMF uses the anatomical structural connectivity matrix to connect the brain regions, following which dynamic interactions Equations 3–9 generate BOLD signals in each parcellated brain region. Finally, the functional connectivity matrix computed from simulated BOLD signal is matched with empirical rs-FC (Figures 1D and 1E).

### Steady-State Solutions of the MDMF Model

In Figures 2A and 2B, relations of GABA- and glutamate-mediated synaptic gating and population mean firing rates are generated by taking the steady-state solutions for the equations (Equations 7 and 8) of the MDMF model, along with GABA- or glutamate-mediated synaptic gating from the model proposed by Destexhe et al. (1994b). We have used fixed values of glutamate (7.46 mmol) and GABA (1.82 mmol) concentrations observed in precuneus of a normal healthy individual’s brain during resting state and reported in a magnetic resonance spectroscopy (MRS) study by Hu et al. (2013). Steady-state synaptic gating from the Destexhe model is computed numerically by integrating Equation 2 for the neurons whose firing statistic was defined by Poisson distribution with mean firing rates from 1 to 300 Hz, and taking the asymptotic value of synaptic gating *S*(*t*) at *t* → ∞. Steady-state values of synaptic gating, $S_{ss}^{E}$ and $S_{ss}^{I}$, for the MDMF model are computed from Equations 7 and 8 analytically (for *σ* = 0) when the system reaches attractor state (setting $\frac{dS}{dt}$ = 0) and the subsequent algebraic steps:

$$S_{ss}^{E}=\frac{\alpha^{E}T_{\text{NMDA}}r^{E}}{\left(\beta^{E}+\alpha^{E}T_{\text{NMDA}}r^{E}\right)}.$$ (16)

$$S_{ss}^{I}=\frac{\alpha^{I}T_{\text{GABA}}r^{I}}{\left(\beta^{I}+\alpha^{I}T_{\text{GABA}}r^{I}\right)}.$$ (17)

Figures 2A and 2B show that in terms of average gating kinetics, the MDMF model results are almost equivalent to the model proposed by Destexhe et al. (1994b). Subsequently, we estimated simulated rs-FCs by solving the system of Equations 3–9 numerically and then computing pairwise temporal correlations among MDMF units, for a range of *G*s for a fixed concentration of glutamate (7.46 mmol) and GABA (1.82 mmol) representative of a healthy brain. Considering optimal scenarios in which FC distance between simulated and empirical rs-FC is minimized (Figure 2C), FC correlation is maximized (Figure 2D), and average firing rate of excitatory populations (Figure 2E) and average firing rate of inhibitory populations (Figure 2F) doesn’t rise above 4 Hz and 9 Hz, respectively, a fixed value of *G* = 0.69 was chosen for all subsequent numerical analysis using MDMF, including the scenarios of diseased brain captured by parametric variation of GABA and glutamate. An important point to note here is that even though *G* is a scaling parameter with no biophysical basis, there can be bifurcations upon parametric variation of *G* (more details are in the Supporting Information). At *G* = 0.69, average firing rate of cortical excitatory populations is guaranteed to be ∼4 Hz, which is within the observed range of mean firing rate of excitatory population in cortex (∼3–6 Hz, Figure 2E; Bittner et al., 2017), and average firing rate of cortical inhibitory populations is ∼9 Hz (Figure 2F). In Figures 2G and 2H, firing rates of excitatory population and inhibitory population, respectively, of each cortical area are shown in all 68 brain areas (the full name of ROIs in each hemisphere is provided in Table 2). The firing rates of excitatory populations across cortical areas are relatively similar, although with some degree of variability as reported previously (Mochizuki et al., 2016).

**Table 2.** All 34 ROIs in each hemisphere. Area ID denotes the order of ROIs in the functional and structural connectivity matrices for each hemisphere

Area ID	Abbreviation	Full name
1.	BSTS	Banks of superior temporal sulcus
2.	CAC	Caudal anterior cingulate
3.	CMF	Caudal middle frontal
4.	CUN	Cuneus
5.	ENT	Entorhinal
6.	FUS	Fusiform
7.	IP	Inferior parietal
8.	IF	Inferior temporal
9.	IC	Isthmus cingulate
10.	LO	Lateral occipital
11.	LOF	Lateral orbitofrontal
12.	LIN	Lingual
13.	MOF	Medial orbitofrontal
14.	MT	Middle temporal
15.	PH	Parahippocampal
16.	PC	Paracentral
17.	PAOP	Pars opercularis
18.	PAOR	Pars orbitalis
19.	PT	Pars triangularis
20.	PEC	Pericalcarine
21.	POCE	Postcentral
22.	POCI	Posterior cingulate
23.	PRCE	Precentral
24.	PRCU	Precuneus
25.	RAC	Rostral anterior cingulate
26.	RMF	Rostral middle frontal
27.	SF	Superior frontal
28.	SP	Superior parietal
29.	ST	Superior temporal
30.	SM	Supramarginal
31.	FP	Frontal pole
32.	TP	Temporal pole
33.	TT	Transverse temporal
34.	INS	Insula

### Determining Local Homeostasis Regime of GABA and Glutamate Concentrations Relating to rs-FC

**Figure 3.**
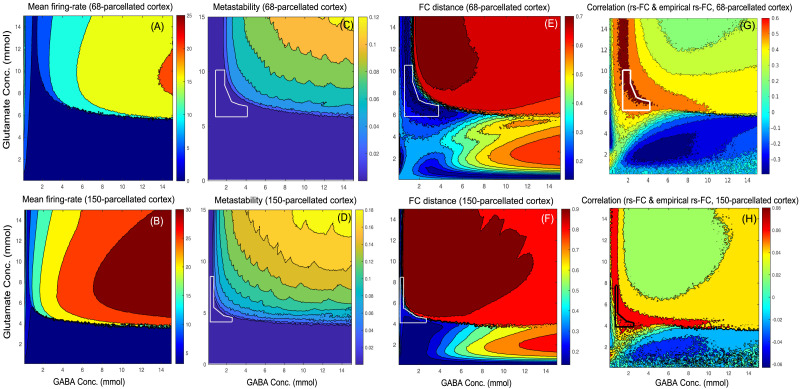
(A, B) Neuronal firing, (C, D) metastability, and (E, F) FC distance as a function of GABA and glutamate concentrations. (A, B) Mean firing rate: Average of firing rates of excitatory population using (A) Desikan-Killiany and (B) Destrieux parcellations. (C, D) Metastability computed from the simulated BOLD signals using (C) Desikan-Killiany and (D) Destrieux parcellations. (E, F) FC distance: Similarity between empirical rs-FC and simulated rs-FC computed using Frobenius norm over various GABA (0.1–15 mmol) and glutamate (0.1–15 mmol) concentrations using (E) Desikan-Killiany and (F) Destrieux parcellations. (G, H) FC correlation: Correlation between empirical rs-FC and simulated rs-FC computed over various GABA (0.1–15 mmol) and glutamate (0.1–15 mmol) concentrations using (G) Desikan-Killiany and (H) Destrieux parcellations.

We solved the system of Equations 3–9 numerically over a parameter space of glutamate and GABA concentrations from 0.1 to 15 mmol. Mean of firing rates of excitatory population across all parcellated cortical areas are examined for various GABA and glutamate concentrations. Two different parcellations—Desikan-Killiany (Desikan et al., 2006) and Destrieux (Destrieux et al., 2010)—are used to evaluate the robustness of the results. Figures 3A and 3B depict mean firing rate of excitatory populations across 68 and 150 parcellated cortical areas, respectively, at glutamate concentration ranging from 0.1 to 15 mmol and GABA concentration ranging from 0.1 to 15 mmol. Metastability is computed using Kuramoto order parameter (see the Materials and Methods section for details) from simulated BOLD signals from parcellated brain areas (Figures 3C, 3D) across various concentrations of GABA and glutamate. Metastability computed from empirical BOLD signals obtained from subsamples of Cam-CAN dataset (40 subjects) is found to range from ∼0.0002 to ∼0.003. So, the region outlined by white lines in Figures 3C and 3D denotes parameter regimes where metastability from simulated BOLD signals closely matches metastability obtained from empirical BOLD signals. Hence, using the Desikan-Killiany atlas, a match between metastability of simulated BOLD signal and empirical BOLD signal is found at glutamate concentrations ranging from 6.1 to 10.5 mmol and GABA concentrations ranging from 1.5 to 4.2 mmol (Figure 3C). Even with a finer parcellation scheme (Destrieux et al., 2010), glutamate concentrations ranging from 4.2 to 9.1 mmol and GABA concentrations ranging from 0.2 to 3 mmol show a good match between metastability of simulated BOLD signal and empirical BOLD signal (Figure 3D). Also, the maximum similarity between the empirical rs-FC and model-predicted rs-FC, measured by the Frobenius norm and correlation, is found at glutamate concentrations ranging from 6.1 to 10.5 mmol and GABA concentrations ranging from 1.5 to 4.2 mmol (Frobenius norm, Figure 3E, and FC correlation, Figure 3G). Using a finer parcellation scheme (Destrieux atlas, 150 parcellated brain areas), the maximum similarity between the empirical rs-FC and simulated rs-FC measured by Frobenius norm and correlation is found at glutamate concentrations ranging from 4.2 to 9.1 mmol and GABA concentrations ranging from 0.2 to 3 mmol (Frobenius norm, Figure 3F, and FC correlation, Figure 3H). Empirical observations from MRS studies show that an adult normal human brain contains glutamate from 6 to 12.5 mmol/kg and GABA from 1.3 to 1.9 mmol/kg (Govindaraju et al., 2000).

Finally, we have argued that the local homeostasis of E–I balance is obtained at glutamate concentrations ranging from 6.1 to 10.5 mmol and GABA concentrations ranging from 1.5 to 4.2 mmol using the Desikan-Killiany atlas. Here, both metastability and FC distance are employed to determine the optimal regime of GABA and glutamate concentrations.

### Relationship Between Neurotransmitter Concentrations and the Degree of Functional Segregation and Integration

**Figure 4.**
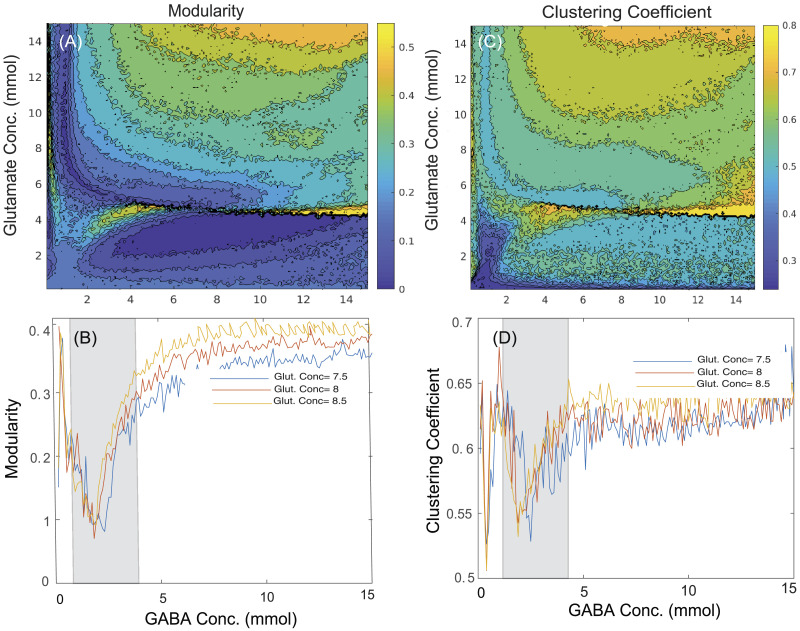
Contour plots showing the changes in network segregation measures such as (A) modularity and (C) clustering coefficient across various concentrations of GABA (0.1–15 mmol) and glutamate (0.1–15 mmol). The effect of changes in GABA concentrations ranging from 0.1 to 15 mmol at discrete values of glutamate concentration (7.5 mmol, blue line; 8 mmol, red line; 8.5 mmol, yellow line) is shown on network segregation measures including (B) modularity and (D) clustering coefficient. The shaded regions denote graph theoretical measures in homeostasis regime of GABA ranging from 1.5 to 4.2 mmol with discrete values of glutamate concentration (7.5, 8, or 8.5 mmol), whereas outside the shaded regions represents pathological scenarios.

**Figure 5.**
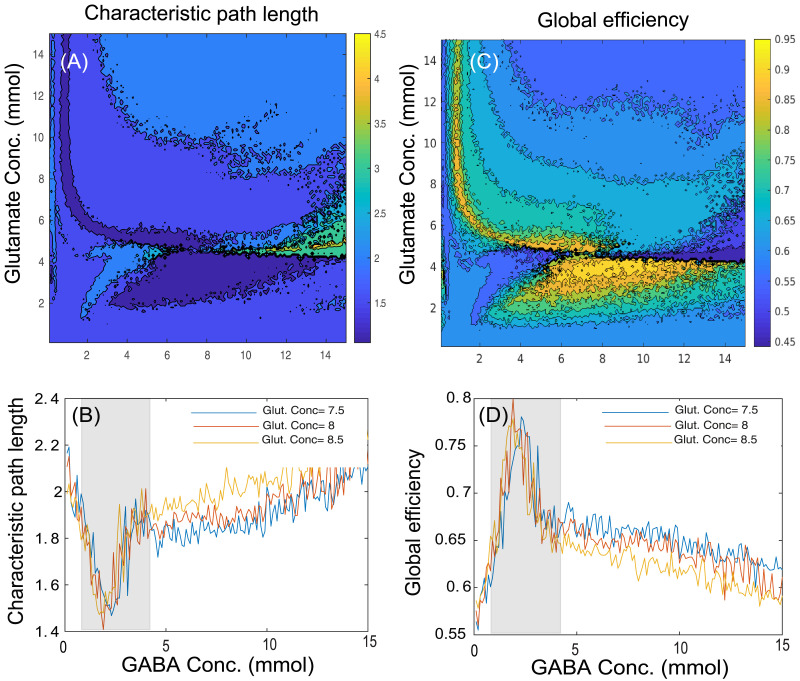
Contour plots showing the changes in network integration measures such as (A) characteristic path length and (C) global efficiency across various concentrations of GABA (0.1–15 mmol) and glutamate (0.1–15 mmol). The effect of changes in GABA concentrations ranging from 0.1 to 15 mmol at discrete values of glutamate concentration (7.5 mmol, blue line; 8 mmol, red line; 8.5 mmol, yellow line) is shown on network integration measures like (B) characteristic path length and (D) global efficiency. The shaded regions denote graph theoretical measures in homeostasis regime of GABA ranging from 1.5 to 4.2 mmol with discrete values of glutamate concentration (7.5, 8, or 8.5 mmol), whereas outside the shaded regions represents pathological scenarios.

In the present study, we have computed the graph theoretical properties that quantitate functional segregation and integration from simulated functional connectivity using the Brain Connectivity Toolbox. Figures 4A and 4C illustrate the network segregation measures, modularity and clustering coefficient, respectively, while Figures 5A and 5C represent network integration measures, characteristic path length and global efficiency, respectively, across various GABA and glutamate concentrations. To represent variation in graph theoretical measures at the specific neurotransmitter concentrations, Figures 4B and 4D represent changes in modularity and clustering coefficient, respectively, whereas Figures 5B and 5D illustrate variations in characteristic path length and global efficiency, respectively, at the discrete concentration of glutamate (7.5 mmol, blue line; 8 mmol, red line; 8.5 mmol, yellow line) over various concentrations of GABA. Here, the homeostasis regime of GABA concentrations ranging from 1.5 to 4.2 mmol are marked by the shaded region.

The interpretations from network segregation measures (Figures 4B and 4D) and integration measures (Figures 5B and 5D) with glutamate–GABA concentrations illustrate the following scenarios of neurotransmitter level: (a) low GABA concentration (0.1–1.4 mmol), (b) homeostasis regime of GABA concentration (1.5–4.2 mmol; shaded region), and (c) high GABA concentration (4.3–15 mmol), along with the discrete concentrations of glutamate (7.5, 8, or 8.5 mmol; selected from the homeostasis regime of glutamate). All the segregation measures (modularity, Figure 4B; clustering coefficient, Figure 4D) have minima for a regime of optimal GABA and glutamate values. On the other hand, the characteristic path length has minima for optimal concentrations of GABA–glutamate (Figure 5B), while the global efficiency peaks at the optimal concentration of GABA and glutamate (Figure 5D).

**Figure 6.**
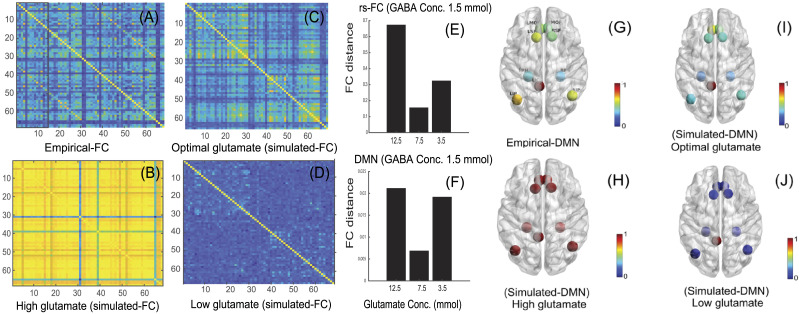
Comparison of empirical rs-FC and simulated rs-FC. (A) Empirical rs-FC. (B) Simulated rs-FC with 12.5 mmol glutamate and 1.5 mmol GABA (high glutamate), (C) 7.5 mmol glutamate and 1.5 mmol GABA (optimal glutamate), and (D) 3.5 mmol glutamate and 1.5 mmol GABA (low glutamate). (E) FC distance between empirical rs-FC and simulated rs-FC obtained at various glutamate concentrations including 12.5 mmol, 7.5 mmol, or 3.5 mmol glutamate with GABA concentration fixed to 1.5 mmol. (F) FC distance between empirical DMN and simulated DMN nodes generated at different glutamate concentrations including 12.5 mmol, 7.5 mmol, or 3.5 mmol glutamate with 1.5 mmol GABA. Pairwise correlation between nodes of DMN with left isthmus cingulate (LIC) as the seed region and left inferior parietal (LIP), left medial orbitofrontal (LMOF), left parahippocampal (LPH), left superior frontal (LSF), right inferior parietal (RIP), right medial orbitofrontal (RMOF), right parahippocampal (RPH), and right superior frontal (RSF) shown at various scenarios: (G) empirical-DMN, (H) high glutamate (12.5 mmol glutamate and 1.5 mmol GABA), (I) optimal glutamate (7.5 mmol glutamate and 1.5 mmol GABA), and (J) low glutamate (3.5 mmol glutamate and 1.5 mmol GABA).

Empirical rs-FC (Figure 6A), simulated rs-FC generated at high glutamate concentration (Figure 6B), optimal glutamate concentration (Figure 6C; homeostatic regime), and low glutamate concentration (Figure 6D) with GABA concentration fixed at 1.5 mmol demonstrate that maximum similarity between empirical and simulated data (Figures 6A and 6C) can be achieved at optimal GABA/glutamate values. Euclidean distance between the simulated and empirical rs-FC is computed using the Frobenius norm (Vattikonda et al., 2016) and is found to be minimum for optimal GABA/glutamate (Figure 6E). In addition, FC distances computed using only default mode network (DMN) nodes indicate high similarity between empirical and simulated data for optimal values of GABA/glutamate (Figure 6F). An earlier report shows that an increased glutamate/GABA ratio results in enhanced correlations among DMN network nodes (Kapogiannis, Reiter, Willette, & Mattson, 2013). Hence, we argue that MDMF can qualitatively predict the reorganization of correlations among large-scale brain networks following changes in neurochemical concentrations. Brain areas of the DMN comprise left isthmus cingulate, left inferior parietal, left medial orbitofrontal, left parahippocampal, left superior frontal, right inferior parietal, right medial orbitofrontal, right parahippocampal, and right superior frontal (Seibert & Brewer, 2011) are all selected from the 68 brain regions distributed in the right and left hemispheres based on the Desikan-Killiany atlas (Vattikonda et al., 2016). Here, left isthmus cingulate is selected as a seed region. In Figures 6G–6J, we have shown how correlation among nodes of DMN changes in various scenarios such as high (Figure 6H), optimal (Figure 6I), or low (Figure 6J) glutamate concentrations as compared with empirical DMN nodes (Figure 6G).

## DISCUSSION

In the present study, we have proposed a multiscale dynamic mean field model that provides a mechanistic explanation of whole-brain resting-state network organization in human brain as a function of underlying change in GABA and glutamate concentrations. In other words, it is an effort to bridge two different scales: neurotransmitter concentration and population activity measured using excitatory firing rate derived from DMF. MDMF brings specificity in precise quantification of the critical range of neurotransmitter concentrations, which allows us to estimate a departure from normative values based on measurement of key network topological properties of integration and segregation. This further opens up the possibility of tracking departures from healthy to pathological brain states in a systematic manner by comparing against literature-derived empirical estimates of the above network measures. The most crucial findings from the current study are the following: (a) demonstration of a regime of optimal balance between neurotransmitter (glutamate–GABA) concentrations in the parameter space and regulation and maintenance of local homeostasis in neural populations in a given brain area; and (b) demonstration of the possibility that both low and high neurotransmitter (glutamate–GABA) concentrations in the parameter space can lead to widely disparate observations of network measures of segregation and integration in clinical studies (we discuss this in detail in later paragraphs). An important caveat of MDMF as well as other biophysically realistic neural models is that even though they give enormous explanatory power and several phenomena can be explained, a direct comparison with other existing models in terms of data fitting is ad hoc in nature. In the rest of this section, we concentrate our discussion on the explanatory role that MDMF may play in understanding neural mechanisms underlying resting-state brain activity in health and disease and what insights can be gained by potential generalization studies in the future.

The MDMF model links two distinct scales of observation and measurement; the steady-state metrics from neurotransmitter kinetics and neural field dynamics contribute harmoniously to give rise to emergent functional connectivity patterns. Recent studies have shown that human brain operates at maximum metastability (Deco et al., 2017) and with the operational principles of local feedback inhibition (Deco, Ponce-Alvarez, et al., 2014). The inhibitory plasticity rule (Hellyer et al., 2016) was employed by clamping the firing rate of cortical excitatory population at ∼3 Hz to achieve a robust parameter space for MDMF, which opens up new avenues in the domain of computational neuropsychiatry. Previous studies have identified that characterization of an optimal E–I neurotransmitter homeostasis regime is critical for understanding the dynamical working point shift associated with mental and neurological disorders (Cabral, Hugues, Kringelbach, & Deco, 2012; Cabral, Kringelbach, & Deco, 2012; Deco, McIntosh, et al., 2014). Thus, MDMF could be used as a computational connectomics tool by clinicians and neuroscientists apart for studying a variety of questions related to neuropsychiatric disorders. Usage of computational whole-rain models in predicting seizure propagation has been recently highlighted by Proix, Bartolomei, Guye, and Jirsa (2017). To validate the applicability of the MDMF model in the diseased brain, we undertook an extensive literature research to identify the changes of glutamate–GABA concentrations in neurological disorders (see Tables 3 and 4).

**Table 3.** Comparison of GABA levels measured with brain MRS recordings from epileptic patients and healthy subjects reported in the literature

Sl. No.	Neurotransmitter	Epileptic patients as compared with control	Brain region	Reference
1.	GABA	Increased	Dorsolateral prefrontal cortex	Chowdhury et al. (2015)
2.	GABA	Decreased	Occipital lobe	Petroff et al. (1995)
3.	GABA	Increased	Frontal area	Hattingen et al. (2014)

**Table 4.** Network segregation measures reported in literature using human brain fMRI from epileptic and schizophrenia patients relative to healthy subjects discrete

Sl. No.	Network segregation measures of brain	Epileptic patients as compared with control	Reference	SZ patients as compared with control	Reference
1.	Clustering coefficient	Increased	Jiang et al. (2017); Pedersen et al. (2015); Wang et al. (2014); Yasuda et al. (2015)	Increased	Hadley et al. (2016)
	Clustering coefficient	—	—	Decreased	Y. Liu et al. (2008); Lynall et al. (2010)
2.	Modularity	Increased	Pedersen et al. (2015)		

Interestingly, deviation from optimal neurotransmitter concentrations can help in prediction of pathological brain network states using MDMF. Graph properties such as modularity, clustering coefficient, global efficiency, and characteristic path length computed from simulated rs-FC could decrease or increase depending on how far away from the optimal glutamate–GABA concentrations are chosen. A close qualitative match between empirical reports of segregation and integration measures obtained from clinical studies and those predicted from the MDMF model was achieved (see Figures 4 and 5). Importantly, the MDMF model may help in conceptualizing the pathophysiology of neuropsychiatric and neurodegenerative disorders, where the communication among brain areas can be classified in terms of local and global measures, and their relationship with the underlying physiological mechanism at the molecular level. In epileptic patients, some studies report increased GABA concentration; in contrast, others report decreased GABA concentration in a brain area as compared with healthy subjects (details of the studies are provided in Table 3). Concomitantly, network segregation measures such as modularity are found to be increased in epileptic patients (Table 4). An increase or decrease in GABA concentration relative to the optimal regime of GABA concentration (shaded region in Figure 4) may result in increased modularity. Hence, the MDMF model could successfully predict how changes in GABA concentration (both an increase or a decrease) relative to optimal GABA concentration lead to increased modularity. Further, previous empirical studies have reported that network integration measures such as characteristic path length are increased in epileptic patients, whereas global efficiency is reported to be decreased (Table 5). Here, the MDMF model could demonstrate how deviations in GABA concentration relative to the optimal regime of GABA concentration (shaded region; refer the Results section) results in increased modularity and a decrease in global efficiency.

**Table 5.** Network integration measures reported in literature using human brain fMRI from schizophrenia and epileptic patients relative to healthy subjects (empirical observations)

Sl. No.	Network integration measures of brain	Epileptic patients as compared with control	Reference	SZ patients as compared with control	Reference
1.	Characteristic path length	Increased	Wang et al. (2014)	Increased	Sun et al. (2016)
2.	Global efficiency	Decreased	Wang et al. (2014); Yasuda et al. (2015)	Decreased	Ganella et al. (2017); Hadley et al. (2016)

In schizophrenia patients, GABA concentrations are found to be lowered in occipital cortex (Thakkar et al., 2017) and prefrontal cortex (Marsman et al., 2014) as compared with healthy subjects (Table 6). On the other hand, network segregation measures such as clustering coefficient are reported to be increased or decreased in schizophrenia patients (Table 4). MDMF shows that a decrease in GABA (0.8 to 1.4 mmol) is associated with an increase in clustering coefficient for the specific regime of GABA concentration. If GABA concentration goes below 0.8 mmol, a sudden drop in clustering coefficient is observed (see the Results section). Therefore, the MDMF model could predict how a decrease in GABA concentration relative to the optimal GABA concentration may result in both an increase or a decrease in network segregation measures such as clustering coefficient. Thus, the most important and unique feature of the MDMF model is that it could link two different parameters such as neurotransmitter concentrations and network measures in a unified modeling framework. We argue that such qualitative predictive power from a biologically realistic model has immense potential to explain interindividual variability of metrics evaluated over large population cohorts of neurological and mental disorders as well as healthy aging. In the future, we would like to extend this model to datasets where the local excitatory–inhibitory homeostasis has been artificially altered, for example after medical drug usage. Such datasets can potentially expand the predictive power of the MDMF model.

**Table 6.** Comparison of GABA levels measured with brain MRS recordings from schizophrenia patients and healthy subjects reported in the literature

Sl. No.	Neurotransmitter	Schizophrenia patients as compared with control	Brain region	Reference
1.	GABA	Decreased	Occipital cortex	Thakkar et al. (2017)
2.	GABA	Decreased	Prefrontal cortex	Marsman et al. (2014)

Although inspired by its success, the MDMF model differs substantially in many ways from the previously proposed DMF model (Deco, Ponce-Alvarez, et al., 2014) that introduced the local excitatory–inhibitory balance. In addition to capturing the essence of modulation in neurotransmitter concentration using the multiscale parameter space of the MDMF model, the inhibitory plasticity rule replaces the feedback inhibition control used in earlier modeling efforts (Deco, Ponce-Alvarez, et al., 2014; Vattikonda et al., 2016) towards regulating homeostatic E–I balance mechanisms. Our results demonstrate that even if the inhibitory plasticity rule is applied locally, it can affect globally, that is, the large-scale brain dynamics. The local inhibitory plasticity rule in the whole-brain network is biologically relevant for providing stabilization in a plastic network and regulating optimal information flow and noise correlation (Vogels et al., 2011). The dynamical consequences of including this feature or that of feedback inhibition control in a network of neural masses has not been well explored, and future work will need to address this limitation. Nonetheless, the steady-state solutions linking GABA/glutamate to synaptic gating provides an ideal entry point to visualize the homeostasis of excitatory–inhibitory currents and neurotransmitters from an unique analytic vantage point.

An important limitation of the MDMF at the current stage is that the modulations in neurotransmitter concentration as a state variable are ignored. All references to the multiscale aspects are limited to the parameter space only. However, the concentrations of these neurotransmitters are variable across different areas; these can be measured from positron emission tomography (PET) recordings (D’Hulst et al., 2015). Nonetheless, a detailed analysis with glutamate–GABA values in individual brain areas contributing to resting-state dynamics remains out of the scope at this point because of lack of availability of detailed data, but it will be an interesting issue to resolve in the future as and when such data are available. Another important limitation is the absence of distance-dependent conduction delays that modulate communication and synchronization between brain areas. To keep our findings tractable and to avoid complexity, delays were neglected in this study; these may serve as a critical ingredient for shaping up global brain dynamics, giving rise to phenomena such as oscillations and other complex spatiotemporal patterns such as chaos and multistability (Deco, Jirsa, McIntosh, Sporns, & Kötter, 2009; Ghosh, Rho, McIntosh, Kötter, & Jirsa, 2008). In addition, we have avoided the incorporation of neuromodulatory effects in the MDMF model because the discussion is purely limited to a relatively small time window of resting-state dynamics. Lastly, the proxy measure of metastability, the standard deviation of the Kuramoto order parameter, that has been used in this article can be modified in the future by incorporating the distributions of higher order moments. However, incorporation of all these features is possible in the MDMF framework in the future. In fact, the DMF component can be replaced with thalamocortical models (e.g., Freyer et al., 2011; van Albada & Robinson, 2009) to address homeostasis in EEG/MEG data in future computational studies.

To conclude, we have characterized the homeostasis of glutamate–GABA concentration via a realistic large-scale neural model in the healthy brain and elucidated how any deviation from the homeostasis regime of neurotransmitter concentrations leads to pathological conditions. The MDMF model could be used to draw inferences from multiple scales of observations of neurotransmitter concentration and neurovascular organization captured by correlations among BOLD time series. Interestingly, the identification of the pathological neurotransmitter concentration space also opens up a novel future direction in the quest of identifying specific sets of biomarkers for characterizing progression from health to disease. Another potentially interesting direction for this approach could be fMRI data during specific sensorimotor and cognitive tasks that are far less traversed at this point. Another future direction of MDMF could be geared more towards generating specific predictions during task conditions or perturbation with brain stimulations, such as transcranial direct stimulation/transcranial magnetic stimulation (tDCS/TMS) with a high degree of patient specificity. Virtual lesions can be introduced as outlined in Vattikonda et al. (2016) for identification of reorganization in the whole-brain connectome as a function of neurotransmitter homeostasis. This remains a target for our future research.

## ACKNOWLEDGMENTS

We acknowledge the generous support of NBRC Core funds and the Computing facility. For simulation of the MDMF model, resources from Neuroscience Gateway (Sivagnanam et al., 2013) were used in the present study. Data collection and sharing for this project was provided by the Cambridge Centre for Ageing and Neuroscience (Cam-CAN). Cam-CAN was supported by the UK Biotechnology and Biological Sciences Research Council (Grant BB/H008217/1), together with support from the UK Medical Research Council and University of Cambridge, UK. In accordance with the data usage agreement for Cam-CAN dataset, the article has been submitted as open access.

## ETHICS STATEMENT

Cam-CAN dataset was collected in compliance with the Helsinki Declaration, and has been approved by the local ethics committee, Cambridgeshire 2 Research Ethics Committee (reference: 10/H0308/50).

## CODE AND DATA AVAILABILITY

Cambridge Centre for Ageing and Neuroscience (Cam-CAN) data are open source and can be downloaded from https://www.cam-can.org/. Codes and relevant data used in this manuscript can be downloaded from https://bitbucket.org/cbdl/mdmf_codes/src/master/ (Naskar, 2021a) and https://osf.io/e58pb/?view_only=5aee8baa772b4607b8f8c3a5a6016fd6 (Naskar, 2021b), respectively.

## SUPPORTING INFORMATION

Supporting information for this article is available at https://doi.org/10.1162/netn_a_00197.

## AUTHOR CONTRIBUTIONS

Amit Naskar: Data curation; Funding acquisition; Investigation; Methodology; Software; Validation; Visualization; Writing – original draft. Anirudh Vattikonda: Data curation; Formal analysis; Investigation; Methodology; Validation; Visualization; Writing – review & editing. Gustavo Deco: Investigation; Methodology; Validation; Visualization; Writing – review & editing. Dipanjan Roy: Conceptualization; Funding acquisition; Investigation; Methodology; Project administration; Resources; Software; Supervision; Validation; Visualization; Writing – review & editing. Arpan Banerjee: Conceptualization; Formal analysis; Funding acquisition; Investigation; Methodology; Project administration; Resources; Software; Supervision; Validation; Visualization; Writing – review & editing.

## FUNDING INFORMATION

Amit Naskar, National Postdoctoral Fellowship (PDF/2016/000378), Science and Engineering Research Board, Department of Science and Technology, Government of India, Award ID: PDF/2016/000378. Dipanjan Roy, Ramalingaswami Fellowship, Department of Biotechnology, Government of India, Award ID: BT/RLF/Re-entry/07/2014. Dipanjan Roy, Department of Science and Technology (DST), Ministry of Science and Technology, Government of India, Award ID: SR/CSRI/21/2016. Arpan Banerjee, Ministry of Youth Affairs and Sports, Government of India, Award ID: F.NO.K-15015/42/2018/SP-V.

## TECHNICAL TERMS

Dynamic mean field (DMF) model: Computational model of neuronal network using ordinary differential equations that captures the macroscopic dynamics of population activity observed in a network of neurons with similar statistical properties. One DMF unit can represent a brain area composed of two groups of neuronal populations with similar statistical properties—one group consisting of excitatory neurons and the other inhibitory, such that interactions among all neurons can be conceptualized as the mean activity across all neurons.
Metastability: A dynamical property exhibited by high-dimensional complex systems, such as the brain. In such systems, a global stable attractor state may not be achieved in the observed timescale, and the systems perpetually traverse various attractor states (also called the metastable states) by switching intermittently. Such switching behavior is empirically observed in BOLD-fMRI time series when functional state transitions occur in resting brain.
Neurotransmitter: Chemical messenger involved in signal transmission from neuron to neuron via synapse.
Blood-oxygen-level-dependent (BOLD) signal: Indirect measure of neuronal activity via detecting changes in blood oxygenation and blood flow in a brain area obtained in functional magnetic resonance imaging (fMRI) scans.
Excitatory–inhibitory balance: Ratio of excitatory neurotransmitter (glutamate) and inhibitory neurotransmitter (GABA).
Multiscale: Refers to techniques that try to relate the activity between two spatiotemporal scales of neuronal organization, such as fast electric signaling in neurons and slow chemical signaling via neurotransmitters.
Network measures: Statistical metrics computed based on topological properties of nodes and edges embedded in a network.
Structural connectome: Map of brain areas connected with physical neuronal connections.
Neurotransmitter homeostasis: A healthy human brain maintains optimal concentrations of neurotransmitters so that excitation and inhibition are balanced to execute normal brain function.
Functional connectome: Map of temporally correlated brain areas based on measured time series activity, such as from BOLD-fMRI, electro/magneto-encephalogram (EEG/MEG).
Magnetic resonance spectroscopy (MRS): Noninvasive technique used to measure metabolic changes such as neurotransmitter concentrations in the human brain.

## External Supplements

https://MDMF_code@bitbucket.org/cbdl/mdmf_codes/src/master/

https://osf.io/e58pb/?view_only=5aee8baa772b4607b8f8c3a5a6016fd6
