# Supplementary material for: Multiscale dynamic mean field (MDMF) model relates resting-state brain dynamics with local cortical excitatory–inhibitory neurotransmitter homeostasis
Source: Netw Neurosci. 2021 Aug 30;5(3):757–82. doi: 10.1162/netn_a_00197 (PMC8567829; doi:10.1162/netn_a_00197)
Supplement: Supplementary file 1 [file netn-05-757-s001.pdf]

Naskar, A., Vattikonda, A., Deco, G., Roy, D., and Banerjee, A. (2021). Supporting information for "Multi-scale dynamic mean field model (MDMF) relates resting-state brain dynamics with local cortical excitatory-inhibitory neurotransmitter homeostasis." *Network Neuroscience*. Advance publication.
https://doi.org/10.1162/netn_a_00197

Supplementary information

We have analysed multiple combinations G, GABA and glutamate to examine good correspondence between simulated and empirical data. However, it could be noted that G (the global coupling between brain areas) is treated here as an ad-hoc scaling parameter that has no biophysical significance as such, and is never changed after fixing it once to overrule any circularity scenarios. We have checked G values for Glutamate and GABA concentration over a range T_{gaba}= 2 mmol, T_{glu} = 7; and T_{gaba}= 1.5, T_{glu}= 6; which captured the reported values in a normal healthy brain. Optimal G varied between 0.51 to 0.74. Hence, G = 0.69 which we chose for all the simulations allows for a fairly reasonable fit with empirical resting state functional connectivity.

We have checked FC correlation and FC distance for other values of G = 0.4 (Fig.S1A & S1B) and G = 0.74 (Fig.S1C & S1D). Since G is a free and ad-hoc parameter, and to be fair any value between 0.51 and 0.74 should work. Accordingly, we demonstrate here the parameter space of FC correlation and FC distance computed for other choice of G values as well.

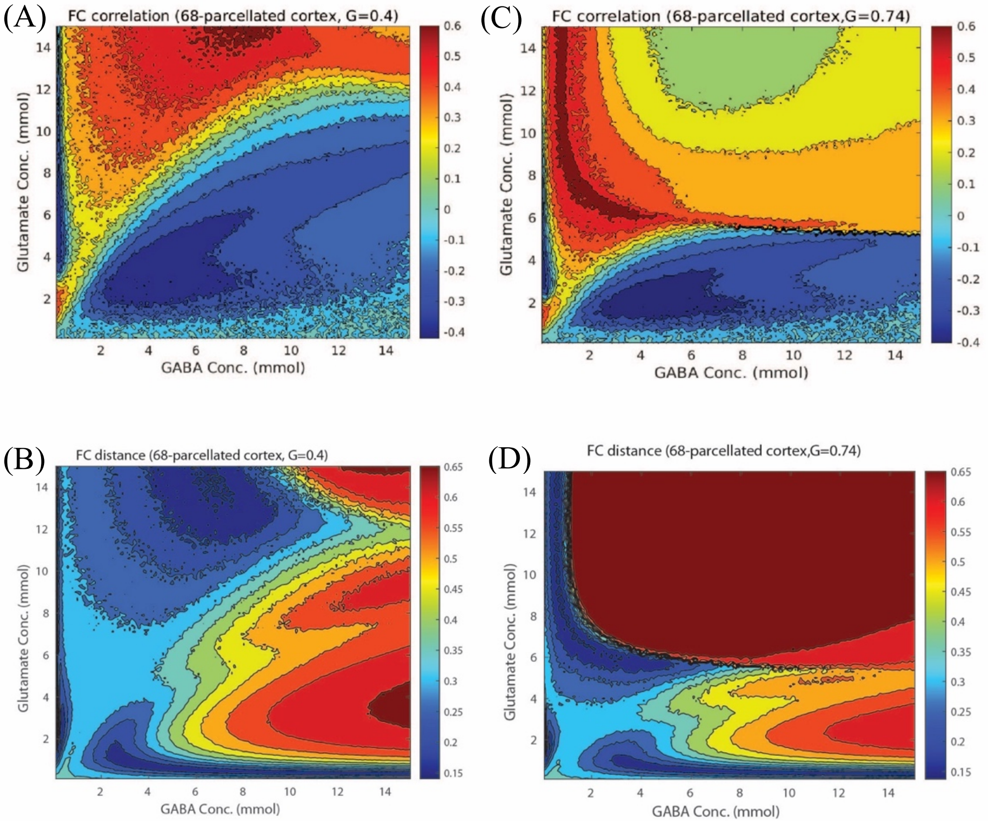

Fig.S1. (A) FC correlation and (B) FC distance as a function of GABA and glutamate concentrations generated at G = 0.4. (C) FC correlation and (D) FC distance as a function of GABA and glutamate concentrations generated at G = 0.74.

To analyse statistical significance, p-values of the Pearson correlation between empirical rs-FC and simulated rs-FC computed can be investigated across various GABA (0.1−15 mmol) and glutamate (0.1−15 mmol) concentrations, for the coarse (Desikan-Killiany atlas, Fig.S2A; 68-parcellated cortex) and fine parcellation scheme (Destrieux atlas, Fig.S2B; 150-parcellated cortex). For both cases, the simulated FC-empirical FC correlation were significant over a wide region of the GABA-glutamate parameter space.

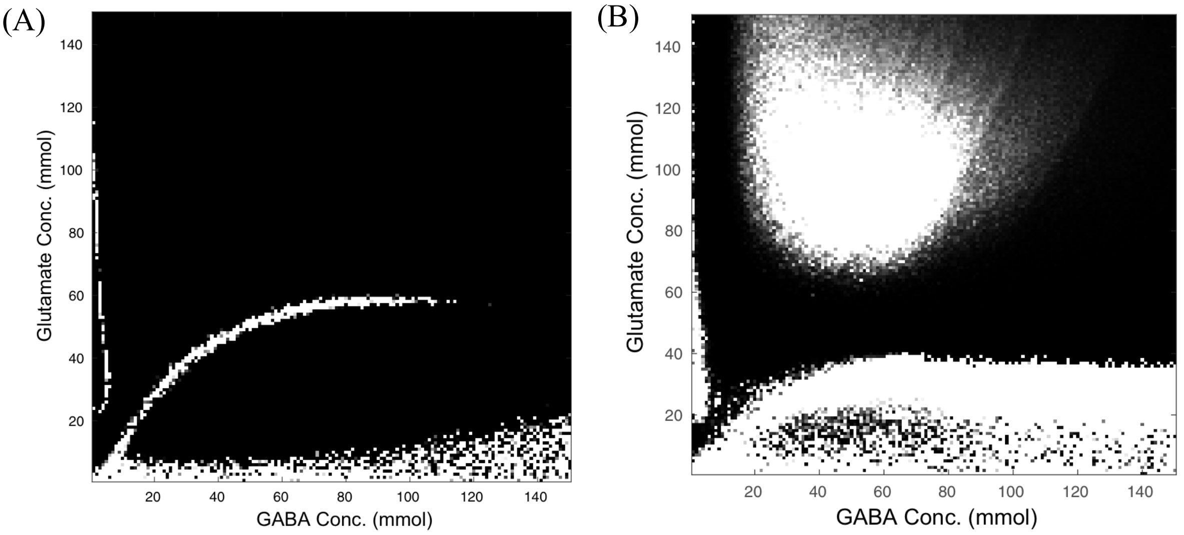

Fig.S2. p-values generated following statistical analysis between simulated rs-FC and empirical rs-FC across GABA and glutamate concentrations. (A) for Desikan-Killiany atlas (68-parcellated cortex) (B) for Destrieux atlas (150-parcellated cortex). In both the cases regions where $p \leq 0.005$, is shaded in black, and $p \geq 0.005$ in white.

To examine the effect of various values of proportion of strongest weight (PSW) on graph theoretical measures, rs-FC matrices are proportional-thresholded with higher or lower values relative to PSW = 0.25. Qualitatively, we have found that graph theoretical measures of functional connectivity across different threshold values show no change as compared to graph theoretical measures of connectivity matrix proportional-thresholded with PSW = 0.25.

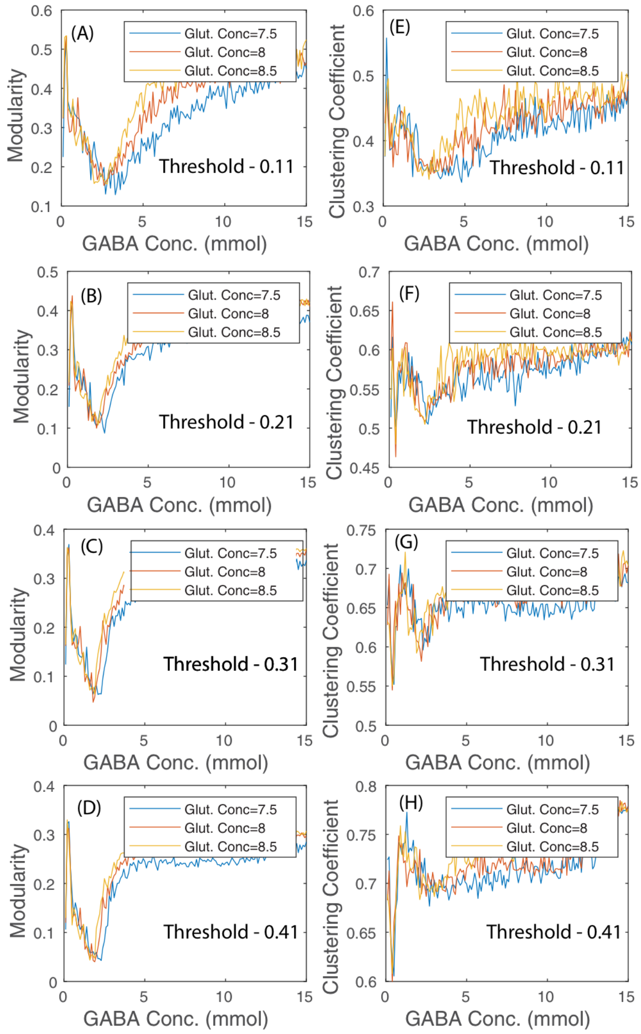

Fig.S3. Effect of various threshold functional connectivity (FC) values on graph theoretical measures. (A) Modularity and (E) clustering coefficients computed from connectivity matrix thresholded with 0.11. (B) Modularity and (F) clustering coefficients obtained from connectivity matrix thresholded with 0.21. (C) Modularity and (G) clustering coefficients measured from connectivity matrix thresholded with 0.31. (D) Modularity and (H) clustering coefficients computed from connectivity matrix thresholded with 0.41.
